# Genome-Wide Transcriptional Response of *Saccharomyces cerevisiae* to Stress-Induced Perturbations

**DOI:** 10.3389/fbioe.2016.00017

**Published:** 2016-02-18

**Authors:** Hilal Taymaz-Nikerel, Ayca Cankorur-Cetinkaya, Betul Kirdar

**Affiliations:** ^1^Department of Chemical Engineering, Bogazici University, Istanbul, Turkey

**Keywords:** yeast, transcriptome, perturbation, regulation, stress

## Abstract

Cells respond to environmental and/or genetic perturbations in order to survive and proliferate. Characterization of the changes after various stimuli at different -omics levels is crucial to comprehend the adaptation of cells to the changing conditions. Genome-wide quantification and analysis of transcript levels, the genes affected by perturbations, extends our understanding of cellular metabolism by pointing out the mechanisms that play role in sensing the stress caused by those perturbations and related signaling pathways, and in this way guides us to achieve endeavors, such as rational engineering of cells or interpretation of disease mechanisms. *Saccharomyces cerevisiae* as a model system has been studied in response to different perturbations and corresponding transcriptional profiles were followed either statically or/and dynamically, short and long term. This review focuses on response of yeast cells to diverse stress inducing perturbations, including nutritional changes, ionic stress, salt stress, oxidative stress, osmotic shock, and to genetic interventions such as deletion and overexpression of genes. It is aimed to conclude on common regulatory phenomena that allow yeast to organize its transcriptomic response after any perturbation under different external conditions.

## Introduction

The central challenge in systems biology is to construct the whole life model for the prediction of cellular response to the changing environments. Therefore, the genome-level understanding of the cellular response to both genetic and environmental perturbations is extremely important in modeling. Consequently, systematic perturbation experiments were conducted to reach this goal. *Saccharomyces cerevisiae* has been investigated in biochemical and genetics laboratories for many decades, since it is considered to be a good model organism. Cellular response of this organism at different -omics levels to different perturbations has also been extensively studied by systematically introducing environmental changes. The availability of a deletion collection made it also an attractive organism to screen the cellular response to deletions of specific genes. The systems biology-based studies resulted in the understanding of several pathways and the cellular behavior of the yeast cells to different perturbations. Although it is probably the best-understood organism, we are still far away from modeling the response of this organism to perturbations (Boone, 2014).

Eukaryotic cells reprogram the expression of those genes that are essential for adapting to the changing conditions as a response to perturbation. Microarray technology allowed investigation of expression profiles of thousands (whole-genome arrays) or hundreds (low-density arrays) of genes simultaneously. ArrayExpress at the European Bioinformatics Institute (EBI) and the Gene Expression Omnibus (GEO) database at the National Center for Biotechnology Information (NCBI) are the two major public databases of microarray data (Barrett et al., 2013; Kolesnikov et al., 2015). Although they have different designs, both databases support the minimum information about a microarray experiment (MIAME), a standard guideline for describing a microarray experiment (Brazma et al., 2001). The establishment of RNA-seq, providing detailed measurement, and lower technical discrepancy, became another attractive analytical tool in transcriptomics (Nagalakshmi et al., 2008; van Dijk et al., 2011; Nookaew et al., 2012). Most of the high-throughput data have been generated by sampling the experimental system at single-time point. Generation of the time-series gene expression data is considered to be very important to understand the dynamic nature of biological systems. The efforts to study and model such dynamic data were reviewed in detail by Bar-Joseph et al. (2012), Yosef and Regev (2011), and Secrier and Schneider (2014).

The programing of gene expression in cells occurs on a broad range of time-scales from rapid responses (minutes to hours of response to environmental stresses) to slower (hours to days during development) processes (Yosef and Regev, 2011). An early analysis of the transcriptomic response of yeast cells to diverse environmental changes indicated that a large set of genes (~900) showed a comparable and severe response to different perturbations sensing them as an environmental stress [environmental stress response (ESR)]. The upregulated genes were related to stress defense regulated by Msn2p and Msn4p and downregulated genes were associated with ribosome biogenesis and protein synthesis. An important observation to be noted was the involvement and the regulation of different isoenzymes in response to several perturbations (Gasch et al., 2000; Causton et al., 2001; Gasch, 2003). Positive and negative regulators of protein kinase A (PKA) pathway were also reported to be induced within the ESR genes (Gasch et al., 2000). This common response to perturbations was reported to be important for preparing cells in response to further possible changes in the environment (Berry and Gasch, 2008; Mitchell et al., 2009) leading to stress (cross-) tolerance. However, it should be noted that the correlation between upregulation of gene expression and its requirement for fitness was not always significant (Giaever et al., 2002; Giaever and Nislow, 2014).

Zakrzewska et al. (2011) used yeast haploid deletion collection to find the genes and their related functions involved in the stress survival and in the gain of stress tolerance. Survival analysis of the yeast cells revealed that a general stress response (GSR) increases survival after a period of mild stress pretreatment, and this survival of stress was negatively correlated with mutant growth rate. Resistance to stress and corresponding tolerance gained, induced by severe perturbations in *S. cerevisiae*, are directed by specific processes for each stress and at the same time by general processes for all stresses. Growth rate was acknowledged for being responsible for tolerating stress and growth rate reduction was found to be responsible for gaining tolerance to stress. Transcriptomic analysis in response to several perturbations in chemostats at different growth rates indicated that there was a notable correlation between the growth rate-dependent genes and ESR genes (Gasch et al., 2000; Regenberg et al., 2006; Castrillo et al., 2007; Brauer et al., 2008; Fazio et al., 2008). It has been proposed that most of the alterations in the expression of ESR genes are associated with the lowered growth rate as a result of a change in the environment. Msn2p and Msn4p are found to be important in the organization of transcriptome, but not in the observed changes, in the phenotype due to changes in the growth rate (Zakrzewska et al., 2011). Components of stress-specific signaling pathways and effectors of these pathways intervening functional adaptation in response to changes in environmental conditions have been identified (Chasman et al., 2014). An extensive review of signaling pathways that control proliferation versus stress defense in yeast and mammalian cells was recently provided (Ho and Gasch, 2015).

Transcriptomic data obtained from perturbation experiments are an extremely important step in the construction of large scale, system-based models that can be used to predict the cellular response to perturbations. The integration of this data with other -omics, pathway information, computational, and statistical tools, is crucial to improve the accuracy in the prediction capabilities of the models. However, it is important to note that these experiments should be carefully designed and interpreted according to the needs of the investigators. In the present review, systematically introduced perturbations to monitor the whole-genome level transcriptomic response of *S. cerevisiae* cells to these perturbations will be summarized. Most commonly studied stress-causing perturbations, such as the changes in the types and quantities of available nutrients, oxidative reagents, temperature, osmolarity, and the metal ions, were selected to review. Moreover, these perturbations are closely related to the optimization of industrial applications and to human diseases. Although we review each perturbation in a different section, it is not always possible to analyze only one stress factor alone. For example, during fermentation, *S. cerevisiae* wine strains undergo considerable stress due to the high concentrations of sugars, producing high osmotic pressure, followed by ethanol accumulation, addition to nitrogen limitation, low pH, and the presence of SO_2_, all imposing pressure on the cells (Treu et al., 2014).

## Transcriptomic Response to Nutritional Changes

The ability of living organisms to acclimate to alterations in their nutritional environment is necessary for their survival, and they have developed mechanisms to cope with the new conditions quickly and effectively. Yeast cells sense the nutrients in the environment *via* a group of major nutrient-signaling pathways, which coordinate general responses, such as cellular proliferation and stress resistance. Systematic perturbation experiments were performed to understand the underlying molecular mechanisms responsible for this adaptation process to the changes in the environment. The response and tolerance to nutrient-relevant stresses have been largely reviewed by Teixeira et al. (2011) and Conrad et al. (2014). Our focus here will be to review the selected studies on the transcriptomic response of *S. cerevisiae* to nutrient limitations and to transient changes in the nutrient environment as well as on the studies aiming to elucidate the nutrient-signaling pathways.

### Response to Nutrient Limitations

Nutrient limitation has drawn the attention of researchers, because microorganisms encounter nutrient limitation in their natural environments and during industrial processes. The first genome-wide transcriptome analysis in *S. cerevisiae* by DeRisi et al. (1997) investigated the response of yeast cells at transcriptomic level during the diauxic shift using microarrays. This pioneering work indicated that the passage from a glucose rich medium to a glucose-depleted medium involves the integration of a number of major signaling and regulatory pathways. These environmental perturbation experiments revealed that glucose depletion leads to the induction of cytochrome *c*-related genes and the genes involved in the TCA/glyoxylate cycle and carbohydrate storage and to the repression of those involved in protein synthesis, including ribosomal proteins, tRNA synthetases, and translation, elongation, and initiation factors. They further investigated the genome-wide transcriptional response to the deletion of *TUP1*, general transcription repressor, and to the overexpression of *YAP1*, transcription activator, to understand the contribution of those individual regulatory proteins to the reprograming of transcriptional response to glucose. These genetic perturbation experiments revealed the group of genes, expression of which was altered by the deletion of these transcription factors (DeRisi et al., 1997). These results demonstrated for the first time that microarray technology is a useful tool for the genome-wide exploration of expression patterns of genes upon environmental and genetic perturbations. Transcriptional alterations resulting from deletion or overexpression of regulatory molecules identified by this approach were used to dissect and characterize regulatory pathways and networks.

Gasch et al. (2000) examined the temporal transcriptomic changes in yeast cells in batch cultures exposed to amino acid starvation or to nitrogen depletion for a period of 6 h and reported the alteration in the expression levels of ESR genes under both conditions. The genes involved in carbohydrate metabolism, detoxification of reactive oxygen species (ROS), cellular redox reactions, cell wall modification, protein folding and degradation, DNA damage repair, fatty acid metabolism, metabolite transport, vacuolar and mitochondrial functions, autophagy, and intracellular signaling were found to be induced and the genes related to growth-related processes and ribosome biogenesis were reported to be repressed. Furthermore, the transcriptomic analysis of the strains carrying deletion of Msn2p, Msn4p, and both of these factors revealed that the majority of ESR genes were under the control of Msn2p and Msn4p. Starvation-specific response of yeast cells was reported to be related to switch from active growth to a growth-arrested state.

The observation of the effect of changing growth rates on the transcriptomic response to changes in the nutrient environment resulted in a shift of mode of fermentations from batch to chemostat with a constant growth rate. Boer et al. (2003) carried out the analysis of the transcriptome of *S. cerevisiae* cells under carbon-, nitrogen-, phosphorus-, or sulfur-limitation, at a dilution rate of 0.1 h^−1^. This study revealed the significant alterations in the expression levels of 31% of genes between at least two growth conditions. The genes involved in the uptake and phosphorylation of glucose, uptake and metabolism of fatty acids and storage carbohydrates, glyoxylate cycle, and gluconeogenesis, uptake and utilization of alternative carbon sources were upregulated under carbon limitation in addition to the induction of the few genes involved in the protection against oxidative stress. The expression levels of the genes involved in the transport such as low or moderate affinity hexose transporters, the genes involved in the glucose repression, cell proliferation, and differentiation were downregulated under glucose limitation when compared with three other conditions. Under nitrogen limitation, the genes involved in the metabolism of nitrogen-containing compound were observed to be upregulated, and the promoter analysis of coregulated genes revealed that Gln3p, Gat1p, Dal80p, and Gzf3p have important roles in the regulation of genes under nitrogen catabolite repression. Under phosphate limitation, the genes implicated in the uptake and metabolism of inositol phosphate, in the phosphate metabolism, and in the process of phosphorylation of metabolites were found to be induced. From promoter analysis, Pho4p was predicted to be the regulator of these events. Under sulfur limitation, the upregulation of the genes associated with the uptake of sulfate and sulfur-containing molecules and with the sulfur assimilation process was observed. Promoter analysis predicted that the transcription factors including Chf1p–Met4p–Met28p complex, Met31p, and Met32p are involved in the regulation of these genes. The genes involved in the glycogen metabolism, in the export of sulfite and in copper transportation were found to be repressed under phosphate limitation, and the majority of these downregulated genes were predicted to be dependent on Msn2p and Msn4p. It has been suggested that the cellular metabolism reorganized to encounter the needs for nutrient-limited growth of the yeast cells (Boer et al., 2003). Wu et al. (2004) have also examined the genome-wide transcriptome of yeast under five (glucose, ethanol, ammonium, phosphate, and sulfate) different nutrient-limited conditions at a dilution rate of 0.1 h^−1^. The genes affected under each condition were identified with the comparison to its corresponding steady state. This study revealed that the genes involved in the TCA cycle, oxidative phosphorylation, and the genes encoding high-affinity glucose transporters were upregulated under glucose-limited condition. The genes involved in glyoxylate cycles, gluconeogenesis, and nitrogen catabolite repression were upregulated under ethanol-limited conditions even in the presence of sufficient levels of nitrogen in the medium. An interesting result from this study was the observation of the activation of iron-associated genes, including *FTH1*, *FET3*, *FRE3*, *FIT2*, and *FIT3*, by glycolysis. The variances between these two investigations may stem from the differences in the strain background, in the experimental conditions, and in the analysis of data.

The relationship between growth rate and response to nutrient limitations draws the attention of many researchers (Castrillo et al., 2007; Boer et al., 2008, 2010; Brauer et al., 2008; Slavov and Botstein, 2011, 2013). Castrillo et al. (2007) have investigated the effect of the growth rate on the transcriptional, metabolic, and proteomic responses to nutritional changes in yeast with a special emphasis on TOR pathway. The experiments were carried out at growth rates of 0.07, 0.1, and 0.2 h^−1^ under four different nutrient limitations (glucose, ammonium, phosphate, and sulfate) in chemostats. Data analysis was performed by comparing the nutrient limited with nutrient sufficient condition in each case. This study revealed a common response to increase the growth rates in yeast at all omics levels under all nutrient limiting conditions. The genes upregulated with the increasing growth rate and independent of the nutritional limitation were found to be involved in the processes related to translation initiation, ribosome biogenesis, protein biosynthesis, RNA metabolism, nucleic acid metabolism, nucleus import/export, and proteasome function. The downregulated genes were implicated in biological processes related to the response to external stimulus, cell transduction, autophagy, homeostasis, response to stress, and vesicle recycling within Golgi. It has been suggested that these groups of genes might be involved in maximizing the efficient use of cellular resources in each limiting condition at each different growth rate. Furthermore, it has been demonstrated in this study that most of the genes regulated with growth rates are also targets of the TOR signaling pathway. The models to explain the regulation of metabolic fluxes of carbon/nitrogen, sulfur/folate, and leucine biosynthesis at the protein level were constructed through the integration of quantitative proteomic and metabolomic data.

Brauer et al. (2008) also studied the relationship between the growth rate, entry into cell cycle, glucose metabolism, and the transcriptomic response to different nutritional limitations (i.e., glucose, ammonium, sulfate, phosphate, uracil, or leucine) in 36 steady-state conditions. The transcript levels of ~27% of all yeast genes were found to be either positively or negatively correlated with growth rate, independent of any nutrient limitation, and provided further support for the results published by Castrillo et al. (2007). The genes involved in ribosomal functions and stress response were found to be positively correlated with growth rate, whereas the genes involved in peroxisomal functions were identified to be negatively correlated with growth rate. The relationship between growth rate and cell-cycle population in G0/G1 phase was also linear independent of limiting nutrient. They have also constructed a linear model, based on the gene expression values, to predict relative “instantaneous growth rate” in both batch and continuous cultures. The observation of the complete consumption of glucose and failure in arresting in cell cycle, when the growth of the auxotrophic strains is limited by the auxotrophic requirements, led researchers to investigate this phenomenon further.

Analysis of the transcriptomic response of auxotropic yeast cells grown in chemostat cultures limited only with their respective auxotrophic requirements, at different growth rates, indicated a decoupling of growth rate response (GRR) from nutrient signaling, and the magnitude of the response was found to be less than that recorded in prototrophs. It has been suggested that growth rate signal is important in the regulation of fermentation/respiration, the GRR, and the cell division cycle (Brauer et al., 2008; Slavov and Botstein, 2013).

### Response to Transient Nutrient Changes

The reorganization of the transcriptomic and/or metabolomic response to transient changes in the nutrient environment was investigated by the impulse-like addition of the limited nutrient into yeast cells grown in chemostats under a specific nutrient limitation. The dynamic adaptation to the changing concentration of the nutrient was followed in time-course experiments after the perturbation of the steady state by the addition of the limited nutrient.

Kresnowati et al. (2006) studied transcriptional and metabolic response of yeast cells within the initial 5 min of the perturbation when aerobic, tightly controlled glucose-limited chemostat cultures were subject to a glucose pulse, and a new insight could be provided for the temporal organizations of metabolic and transcriptional events. This study provided the first example of the integration of transcriptome with metabolic changes during the initial response (minutes) to a well-defined perturbation of *S. cerevisiae* cells. They have shown that although metabolic and transcriptomic data are well correlated, the changes in metabolite concentrations were observed in seconds, and the changes in the transcriptomic response were detected later. Cells have responded to this increasing glucose level at transcriptional and metabolic level in order to adapt to the change from respiratory to respiro-fermentative metabolism and to growth acceleration. The same group has also studied the long-term response of yeast cells to a glucose pulse and depletion of oxygen by investigating the dynamic adaptation of *S. cerevisiae* grown in aerobic, glucose-limited chemostat cultures to an anaerobic pulse of glucose for a longer period of time (5, 10, 30, 60, and 120 min). The fact that one-third of the genes were significantly and differentially expressed indicated an important reprograming and strong response of the cells after the pulse. Most of these genes were found to be related to growth and carbon catabolite repression. The expression levels of several genes, including the genes encoding ribosomal biogenesis and ribosomal proteins, were changed in opposite direction 30 min after the pulse (van den Brink et al., 2008).

Bradley et al. (2009) have also shown the coordinated changes between the transcriptome and metabolome by analyzing the dynamic transcriptomic and metabolomic response of yeast cells grown on filters to glucose and ammonium starvation and developed an integrative approach based on Bayesian framework to predict metabolite–transcript correlations.

Dikicioglu et al. (2011, 2012) investigated the transcriptional response in the short (seconds) and long term (hours), by introducing a glucose pulse to a glucose-limited culture and an ammonium pulse to an ammonium-limited culture of yeast cells. The integration of the transcriptomic data from these perturbation experiments with two different network-based approaches presented further information about the time-based regulation of transcriptional reorganization of yeast cells under these conditions. Integration of this transient transcriptome data with the corresponding metabolome data combined with metabolic pathway information revealed the whole-genome level long-term reorganization of the yeast cells. The changes in the transcriptome and metabolome from an initial limited state, followed by a sudden pulse and then by returning to the limited state were identified for the first time in this study. The transient organization of the *de novo* biosynthetic pathways and salvage pathways under these conditions was reported through integration of transcriptome and metabolome.

### Nutrient Signaling

The sensing and signaling networks that direct the transcriptional and metabolic reorganization in response to the changing nutritional conditions were subject to several studies and reviewed largely by Zaman et al. (2008). In order to understand the regulation of transcriptional response of yeast cells to glucose, the transcriptomic data of the selected yeast strains that carry conditional alleles in response to glucose perturbation were analyzed. The integration of genetic knowledge with microarray analysis revealed the presence of five interlocking pathways in glucose signaling. PKA and PKB (Sch9p) were found to be the main regulators, whereas Snf1 and Rgt pathways have restricted roles in the regulation of the transcriptomic response to glucose. A schematic model illustrating the glucose signaling network was constructed. The perception of the environmental nutrients by the cell was reported to be the main determinant of growth rate-dependent organization of transcriptomic response (Zaman et al., 2009).

Livas et al. (2011) investigated the genome-wide transcriptional response of wild-type yeast cells and two suppressor strains lacking PKA activity when grown on ethanol and exposed to glucose for 30 min. This study revealed the set of genes, which are induced or repressed by glucose either dependent or independent of PKA activity. Induction of the genes involved in glucose metabolism and repression of the genes involved in the utilization of alternative carbon sources were found to be exclusively controlled by other pathways and independent of PKA. The genes involved in amino acid synthesis were found to be regulated by PKA, and the response to glucose is transient. The genes regulated by redundant PKA-dependent and PKA-independent signaling pathways included the genes encoding glucose transporters, ribosomal proteins, and the genes involved in the utilization of non-fermentable carbon sources. A fourth group of genes was found to be regulated by the cooperative signaling pathways, including PKA. This study provided additional support that PKA signaling is an important player in the reorganization of the transcriptomic response of *S. cerevisiae* to glucose.

Hughes Hallett et al. (2014) examined the TOR kinase complex I (TORC1) pathway, which has a key role in the regulation of cell growth, by investigating the changes in the yeast transcriptome in response to various perturbations, including glucose starvation and nitrogen starvation for 20 min. Under glucose starvation, PPA2 branch of TORC1 pathway has a low level activity, whereas Sch9 branch was completely switched off. The transcriptomic analysis of *SNF1* deleted strains indicated that cAMP activated kinase (Snf1p) represses TORC1–Sch9 signaling but hyper-inactivates TOR–PPA2 signaling indicating that Snf1p regulates the TORC1 pathway. However, the presence of additional factors remains to be elucidated. Under nitrogen starvation, TORC1–Sch9 signaling was found to be inactive, whereas TORC1–PPA2 signaling has a high activity. The analysis of the corresponding deletion strains showed that the regulators of the TORC1, Gtr1p/Gtr2p, Npr2p/Npr3p, and Rho1p have important roles in nitrogen or amino acid starvation.

In a recent study, Oliveira et al. (2015) investigated the dynamic regulation of nitrogen metabolism by TORC1 pathway in yeast cells by analyzing transcriptome, proteome, and metabolome data. Codesigning of a perturbation matrix to follow the changes at different omics levels and the use of probabilistic model-based analysis that incorporates the prior knowledge resulted in the identification of putative targets and inputs of TORC1 including a novel putative glutamine signal.

In order to understand the pleiotropic role of the PAS kinase Rim15p in the integration of nutrient-signaling networks, such as TOR, PKA, and the Pho80–Pho85 kinase pathways, the transcriptomic responses of prototrophic *RIM15* deletion mutant and a congenic *RIM15* reference strain of *S. cerevisiae* were comparatively analyzed. Cells were grown under severe calorie restriction in anaerobic retentostat cultures near non-growing conditions. This study revealed the important function of Rim15p in cell-cycle arrest and in the integration of nutrient sensing and signaling pathways under nutrient-depleted conditions (Bisschops et al., 2014).

The details of all these nutritional perturbation experiments in *S. cerevisiae* cells are summarized in Table 1. Many aspects of the response to limitation of C-, N-, S-, and P-sources could be revealed, and the details of the growth-regulated response could be elucidated as well as a wealth of information about transcriptional reorganization in response to the changing nutritional environment that was provided by the above mentioned studies. It is important to keep in mind that the information coming from different studies is far away from being integrated into a model due to the differences in the strain background or in the experimental design. Moreover, the use of different algorithms to analyze and interpret the data provokes an additional difficulty in the comparison or integration of these findings. The challenge continues to identify the nutrition signaling pathways by specifically designed experiments on the basis of these observations. It is important to design an experimental platform to analyze and integrate the response to nutritional changes at different omics levels from wild-type as well as from specifically selected strains, based on the previous knowledge.

**Table 1:** **Nutritional perturbation experiments in *S. cerevisiae* cultures**.

Perturbation	Experiment type	Exposure (sampling) time	Measurement technique	Reference
**Nutrient limitations**				
From glucose rich to glucose-depleted medium (Diauxic shift)	NA	From 9 to 21 h	cDNA microarrays	DeRisi et al. (1997)
From glucose grown to amino acid starvation	Shake flasks	Sampled at early exp. phase (0), 0.5, 1, 2, 4, and 6 h	cDNA microarrays	Gasch et al. (2000)
From glucose grown to amino acid starvation + nitrogen limitation		Sampled at early exp. phase (0), 0.5, 1, 2, 4, 8, 12 h, 1, 2, 3, and 5 days		
Aerobic carbon, nitrogen, phosphorus, or sulfur limited	Chemostat at 0.1 h^−1^	Sampled at steady state	Affymetrix Yeast Genome S98 Array	Boer et al. (2003)
Glucose, ethanol, ammonium, phosphate, or sulfate limited	Chemostat at 0.1 h^−1^	Sampled at steady state and starvation phase	Arrays supplied by Hoheisel	Wu et al. (2004)
Carbon, nitrogen, phosphorus, or sulfur limited	Chemostat at 0.07, 0.1, and 0.2 h^−1^	Sampled at steady state	Affymetrix Yeast Genome S98 Array	Castrillo et al. (2007)
Glucose, ammonium, sulfate, phosphate, uracil, or leucine limited	Chemostat at 0.05, 0.1, 0.15, 0.2, 0.25, and 0.3 h^−1^	Sampled at steady state	Agilent Yeast Oligo Microarray (V2)	Brauer et al. (2008)
Histidine-, lysine-limited	Chemostat at 0.05, 0.1, 0.15, 0.2, 0.25, and 0.3 h^−1^	Sampled at steady state	Agilent Yeast Oligo Microarray	Slavov and Botstein (2013)
**Transient nutrient changes**				
Glucose (1 g/L) pulse to glucose-limited	Chemostat at 0.05 h^−1^	0, 0.5, 1, 2, 3.5, 5, and 5.5 min	Affymetrix Yeast Genome S98 Array	Kresnowati et al. (2006)
Anaerobic glucose (200 mM) pulse to aerobic glucose-limited	Chemostat at 0.1 h^−1^	0, 5, 10, 30, 60, and 120 min	Affymetrix Yeast Genome S98 Array	van den Brink et al. (2008)
Switch to glucose-free or ammonium-free medium	Filter-cultures	Sampled at exp. phase (0), 10, 30, 60, 120, 240, and 480 min	Agilent Yeast Oligo Microarray (V2)	Bradley et al. (2009)
Glucose (2% w/v) pulse to glucose-limited and ammonium (0.3% w/v) pulse to ammonium-limited	Chemostat at 0.1 h^−1^	Sampled at 0, 20, 40, 60 s, 8, 16, 24, 32min, 1, 2, 3, 4, 5, and 7 h	Affymetrix Yeast Genome 2.0 Array	Dikicioglu et al. (2011, 2012)
**Nutrient signaling**				
Galactose (2%) or glucose (2%) addition to glycerol grown cells	NA	Sampled at OD_600_ = 0.25 (0), 20, 40, 60, and 80 min	Agilent Yeast Oligo Microarray	Zaman et al. (2009)
Glucose (4%) addition to ethanol grown cultures	NA	Sampled at early exp. phase (0), and 30 min	Affymetrix Yeast Genome S98 Array	Livas et al. (2011)
Switch to glucose, nitrogen, or amino acid-free medium	Shake flasks	Sampled at OD_600_ = 0.60 (0), 20 min	Agilent Yeast Oligo Microarray (V2)	Hughes Hallett et al. (2014)
		Sampled at steady state		
Glutamine (400 mg/L) pulse to proline grown cultures from proline + glutamine grown cultures to glutamine depletion	Batch reactor	Sampled at 0, 3, 7, 10, 14, 24, 56, and 120 min	Affymetrix Yeast Genome 2.0 Array	Oliveira et al. (2015)
Anaerobic glucose limited	Chemostat at 0.025, 0.05, 0.1 h^−1^	Sampled at steady state	Affymetrix Yeast Genome S98 Array	Bisschops et al. (2014)
	Retentostat	Sampled at 2, 9, 16, and 20 days		

## Transcriptomic Response to Oxidative Perturbations

Exposure to oxidative agents causes the production of ROS, which are known to cause severe cellular damage and be involved in cellular processes, such as aging and apoptosis, as well as in the molecular pathogenesis of several severe disorders. The detailed information on cellular antioxidant systems, which protect yeast cells against ROS accumulation, can be found in the reviews (Farrugia and Balzan, 2012; Morano et al., 2012). Oxidative agents used to investigate oxidative stress response (OSR) are hydrogen peroxide, lipid hyperoxides, organic hydroperoxides, such as cumene hydroperoxide (CHP), linoleic acid hydroperoxide, superoxide anion, and heavy metals, such as Fe^2+^ and Cd. A systematic analysis of yeast deletion mutants revealed that the genes involved in the OSR, induced by different agents, consists of a set of genes known as “core genes,” which are observed under a wide variety of oxidative stress condition and another group of genes known as the “oxidant specific” genes (Thorpe et al., 2004). Several perturbation studies were carried out to investigate the dynamic transcriptomic response of yeast cells to oxidative stress caused by different oxidative agents. Since the hydrogen peroxide is a natural ROS as a by-product of the aerobic metabolism and was most extensively used as a model system for oxidative stress, we will mainly focus in this review on the studies where the oxidative stress was induced by hydrogen peroxide treatment.

The dynamic transcriptomic response to the addition of hydrogen peroxide and menadione into batch cultures was first investigated by Gasch et al. (2000) among other environmental stresses for a period of 2–3 h. The genes encoding superoxide dismutases, gluthatione peroxidases, thiol-specific antioxidants, thioredoxin, thioredoxin reductases, glutaredoxin, and glutaredoxin reductase in addition to ESR genes were found to be specifically upregulated in response to both chemicals. Transcriptomic response of *YAP1* deleted yeast strain indicated that Yap1p is an important regulator of the OSR. Causton et al. (2001) have also investigated the transcriptomic response of yeast cells to hydrogen peroxide similarly and reported the specific upregulation of *ROX1*, which is a repressor of hypoxic genes. Comparative transcriptomic analysis of the wild-type, *yap1*Δ, *yap2*Δ, *yap1*Δ *yap2*Δ, yeast cells after treatment with hydrogen peroxide showed that these proteins are regulators of different biological processes in OSR (Cohen et al., 2002).

TOR kinase complex I pathway is known to be involved in the response to a vast variety of stresses (Loewith and Hall, 2011). Hughes Hallett et al. (2014) examined the TORC1 pathway process information by investigating transcriptional reorganization of yeast cells in response to various perturbations, including oxidative stress induced with hydrogen peroxide using an integrative approach. The analysis of two different modules indicated that the genes in the TORC1–Sch9 pathway were downregulated and the expression levels of the genes in the TORC1–PP2A were not or little affected under oxidative shock. The investigation of the phosphorylation levels of the proteins regulated by PP2A by bandshift assay showed that Npr1p and Gln3p are dephosphorylated but Nnk1p remained phosphorylated upon oxidative stress. These results indicated that TORC1–PP2A-branch signaling is weak or moderate under oxidative stress. Furthermore, the analysis of three yeast strains that stop the transmission of the signal from Npr2p/Npr3p, Gtr1p/Gtr2p, and Rho1p to TORC1–Sch9 signaling and *SNF1* deleted strains resulted in the observation that these proteins do not have any effect on this signaling under oxidative stress as well as under osmotic and heat stresses.

Investigation of the dynamic transcriptional response of yeast cells to oxidative stress induced by the addition of CHP at mid-exponential phase under fully controlled conditions revealed early transcriptional events (Sha et al., 2013). Approximately 54% of the genes that are regulated by Msn2p/Msn4p were also found to be significantly and differentially expressed after the treatment with CHP. *YAP1* was found to be upregulated after 6 and 20 min of induction and 52% of its targets were differentially and significantly expressed upon oxidative stress. *HMS2*, *MET28*, *YAP5*, *NUT2*, *ROX1*, and *SUT2* encoding transcriptional factors were also found to be induced during the early response within the first 6 min after the addition of CHP. *MET1*, *MET12*, *MET16*, *MET22*, *MET3*, *MET8*, *CYS3*, and *STR3* regulating sulfur metabolism, which are targets of *MET28*, were upregulated within 20 min. Other members of the YAP family (*YAP3*, *YAP5*, and *YAP7*) were also upregulated in the early response to CHP. Drug resistance-related proteins, the proteins involved in cell wall and cytoskeleton metabolism, and another group of genes of unknown function were found significantly induced within 6 min after the stress induction and returned immediately to their basal levels. A transient repression of the genes involved in cell growth, DNA replication, transcription, and translation was observed within this interval. The genes associated with mitochondrial function and vesicle trafficking were also transiently downregulated in this early period. The transcript levels of the genes that are involved in gluthatione, glutaredoxin, and thioredoxin systems and the genes encoding ROS removing enzymes were induced within this early period. The genes encoding the enzymes of the oxidative branch of the pentose phosphate pathway were upregulated, whereas the branch leading to the synthesis of nucleic acids was repressed. The transcription factor Rpn4p that is regulator of the synthesis of the proteasome subunits was induced earlier than the genes encoding of these subunits to cope with the removal of the accumulated oxidized proteins. A comparative analysis of the results with previous studies (Gasch et al., 2000; Causton et al., 2001), whereas the hydrogen peroxide was used to induce the oxidative stress, revealed the induction of the ESR genes, and the genes involved in the glutathione metabolism and the pentose phosphate pathway. The biological processes associated with transcription and translation were downregulated in all three studies. The induction of cell wall and membrane was specific for the antioxidant CHP. The genes associated with mitochondrial processes were downregulated in response to CHP, whereas upregulated in response to hydrogen peroxide (Sha et al., 2013).

In a recent study, Zhao et al. (2015) investigated the transcriptional response of a strain, which has higher peroxide tolerance ability to 2 mM hydrogen peroxide exposure in comparison to that of control strain. The genes involved in carbohydrate metabolism, fatty acid degradation, glycolysis/gluconeogenesis, peroxisomal matrix, pyruvate metabolism, amino acid metabolism, and nucleotide repair pathways were found to be significantly and differentially expressed between two strains in response to hydrogen peroxide. MAP kinase and cAMP–PKA signaling pathways, which were significantly enriched by the genes responsive to oxidative perturbation, were identified to be involved in the oxidative tolerance of the mutant strain.

A whole-genome scale analysis at different omics levels revealed that Slf1p, which is La-related protein, is involved in the translational control of oxidative stress induced by hydrogen peroxide (Kershaw et al., 2015). Deletion and mutated strains of *SLF1* were used in this study and cultures were treated with hydrogen peroxide for 10 or 60 min to induce oxidative stress.

The details of all these oxidative perturbation experiments in *S. cerevisiae* cells are summarized in Table 2. These studies, focused on the response of *S. cerevisiae* to oxidative stress, revealed a set of regulators including Yap1p and its homologs, Skn7p, Msn2p, and Msn4p and their selective targets. However, construction of a quantitative model, which incorporate sensing, signaling, and regulation, could not be constructed. It should also be noted that further studies are required to identify missing information coming from a unique experimental platform and carefully designed perturbation experiments.

**Table 2:** **Oxidative perturbation experiments in *S. cerevisiae***.

Perturbation	Experiment type	Exposure (sampling) time	Measurement technique	Reference
Hydrogen peroxide (0.30 mM) addition at early exp. phase	Shake flasks	0, 10, 20, 30, 40, 50, 60, 80, 100, and 120 min	cDNA microarrays	Gasch et al. (2000)
Hydrogen peroxide (0.40 mM) addition at mid-exp. phase	Shake flasks	0, 10, 20, 40, 60, and 100 min	Affymetrix YE6100 gene chips	Causton et al. (2001)
Hydrogen peroxide (0.6 mM) addition at mid-exp. phase	NA	1 h	Oligonucleotide arrays	Cohen et al. (2002)
Hydrogen peroxide (2 mM) addition at mid-exp. phase	Shake flasks	0, 20 min	Agilent Yeast Oligo Microarray (V2)	Hughes Hallett et al. (2014)
CHP (190 μM) addition at mid-exp. phase	Batch bioreactor	0, 3, 6, 12, and 20 min	Affymetrix Yeast Genome S98 Array	Sha et al. (2013)
Growth with 2 mM hydrogen peroxide	Shake flasks	Sampled at mid-exp. phase	RNA-sequencing	Zhao et al. (2015)
Hydrogen peroxide (0.4 mM) addition at mid-exp. phase	NA	0, 15, and 60 min	RNA-sequencing	Kershaw et al. (2015)

## Transcriptomic Response to the Changing Temperatures

Yeast cells encounter rapid and large differences in temperature in nature, and industrial strains are being optimized to grow at certain temperatures, depending on the production process. Therefore, in order to understand the mechanisms for adapting to and showing tolerance to different temperatures, perturbation experiments were designed and performed. The adaptation of wine strains to cold is also important to improve the aroma of the wine. Yeast strains were stored at freezing temperatures, and it is critical to comprehend storage temperature effects on the viability/physiology of the cells. Therefore, the transcriptional response of yeast cells to different temperatures was extensively studied.

### Heat Shock or Adaptation to Higher Temperatures

Temporal genome-wide transcriptional changes of wild-type and *MSN2/MSN4* deleted yeast cells in response to a temperature shift from 25 to 37°C was first investigated by Gasch et al. (2000). The changes in the expression levels of ESR genes were observed within the first minutes after the heat shock, and the majority of these genes were found to be regulated by Msn2p/Msn4p by the investigation of the transcriptomic response of double deleted mutants to heat shock. The genes involved in protein folding chaperons were observed to be induced later. Analysis of the transcriptomic response to a similar temperature shift has also provided further support for these findings (Causton et al., 2001).

A comparative genome-wide analysis including the transcriptional analysis of wild-type and *rpd3*Δ strains grown at 25 and 39°C for 20 min resulted in the finding that RpdL3 histone deacetylase complex is involved in the partial regulation of gene expression upon heat exposure. This study indicated the important role of the chromatin modifications in the reorganization of transcriptomic response to the heat exposure. Hsf1p and Msn2p/Msn4p are main regulators of the heat shock, and this complex has a role in the activation of the genes regulated by Msn2p/Msn4p (Ruiz-Roig et al., 2010).

Mensonides et al. (2013) investigated the transcriptome of *S. cerevisiae* in response to a temperature shift from 28 to 41°C to understand the adaptation of yeast cells to high temperature bioprocesses, over a period of 6 h, in batch cultures. In the initial response during the first hour, in which the cell growth was impaired, genes involved in energy metabolism, trehalose metabolism, the genes encoding molecular chaperones were most significantly induced, and the genes coding for components of translation and transcription machinery were provisionally downregulated. Biological processes related to amino acid biosynthesis, nucleotide metabolism, ion transport, and rRNA biosynthesis were found to be induced after 60 min within the cell growth permissive period. The upregulation of stress responsive genes was also observed during this period except the genes involved in trehalose metabolism. Transporters and the genes associated with purine metabolism were downregulated. The genes involved in the PKC1 pathway, also known as cell wall integrity pathway, were found to be upregulated upon heat shock and remained active for about 1 h during heat exposure.

Hughes Hallett et al. (2014) investigated the effect of the heat stress on the TORC1 pathway by exposing yeast cells to 42°C for 20 min. The response was similar to the response of yeast cells to oxidative stress; TORC1–Sch9 signaling was blocked, whereas TORC1–PP2A-branch signaling was weak or moderate.

### Cold Shock or Adaptation to Cold

Acclimatization/adaptation of yeast cells to cold was also extensively studied through perturbation experiments. First perturbation experiments to investigate the dynamic transcriptomic response of yeast cells to a temperature shift from 37 to 25°C were carried out by Gasch et al. (2000). Unlike to the response observed in a temperature shift from 25 to 37°C, the ESR genes were repressed under this condition and showed a very rapid transition to steady-state characteristics at 25°C.

Sahara et al. (2002) performed perturbation experiments to analyze the dynamic transcriptional response in wild-type yeast cells upon exposure to 10°C for 8 h. Genes involved in rRNA synthesis and the biosynthesis of ribosomal proteins were upregulated in the early phase within 30 min and in the middle phase within 2 h, respectively. General stress genes, including the genes involved in trehalose and glycogen biosynthesis, were observed to be induced in the late phase. Additionally, data indicated that cAMP–PKA pathway might have a role in the regulation of these genes. In another study aiming to investigate the adaptation of yeast cells to cold, wild-type and *MSN2/MSN4* deleted yeast cells were exposed to 10°C for varied time periods changing up to 60 h. This study has also provided additional support that ESR genes, including the genes encoding various heat shock proteins and gluthatione/glutaredoxin system, were upregulated during late cold response, which is dependent on Msn2p/Msn4p. The genes involved in RNA metabolism and lipid metabolism were induced during early cold response which was Msn2/Msn4 independent (Schade et al., 2004).

The analysis of the transcriptomic response of yeast cells grown in batch cultures at 25°C and exposed to near-freezing temperature for different periods of time ranging from 6 to 48 h revealed that the genes involved in trehalose and glycogen synthesis and the genes encoding phospholipids, mannoproteins, cold shock proteins, heat shock proteins, and glutathione were upregulated for cold adaptation. The downregulation of the genes involved in protein synthesis at 4°C is in agreement with the observed delay in growth (Murata et al., 2006).

Tai et al. (2007) studied the genome-wide expression of *S. cerevisiae* cells in response to suboptimal temperatures at steady state. Yeast cells were grown in anaerobic glucose-limited and ammonium-limited chemostat cultures at a dilution rate of 0.03 h^−1^, at 12 and 30°C. At low temperature (12°C), transcription levels of ribosome-biogenesis genes were increased, and in contrast to batch cultures, the expression levels of 88% of ESR genes were decreased. A group of genes involved in nuclear export and ribosome biogenesis was found to be upregulated and the genes involved in carbohydrate metabolism, transport, and response to stimulus were downregulated under both nitrogen and carbon limited conditions. This study revealed adaptational differences between the long-term exposure and a rapid shift to low temperature pointing out no need for trehalose and glycogen for the cold adaptation at steady state.

Comparative transcriptomic analysis of the effect of the low temperature (15°C), between a laboratory and a wine strain grown under anaerobic nitrogen-limited conditions in chemostats resulted in the identification of strain-specific and temperature-dependent genes. The absence of induction of the genes mediated by stress response elements implied that the GSR was repressed under 15°C in comparison to 30°C. The genes involved in trehalose metabolism and in GSR were found to be downregulated, and the genes involved in ribosome biogenesis, RNA processing were upregulated at low temperature in both strains. Integration of the transcriptome with the metabolic topology indicated that glycogen metabolism, amino acid transport, glycolipid biosynthesis, arginine biosynthesis, and allantoin metabolism were affected by the temperature. The transcript levels of the genes involved in sugar uptake and nitrogen metabolism, and transcript levels of genes related to organoleptic properties were significantly different between the two strains. The expression level of *HSF1*, encoding a heat shock transcription factor, which is active under diverse stress conditions, was lower at 15°C in both strains (Pizarro et al., 2008). García-Ríos et al. (2014) also investigated the adaptation of two different wine strains, grown in chemostat cultures at 0.028 h^−1^, to 15°C in order to improve the wine aroma. The integrative analysis of transcriptome with metabolome and proteome data revealed the upregulation of the sulfur assimilation pathway and glutathione biosynthesis during adaptation to cold and the response to low temperature was found to be strain specific.

The details of all these perturbation experiments in *S. cerevisiae* cells are summarized in Table 3. One of the common observations was that the set of the genes affected by temperature up- or downshift was different in the early and late phases of the perturbation in batch cultures. *MSN2*/*MSN4*-dependent ESR genes were found to be upregulated in the late phase during temperature downshift and in the early phase during the temperature upshift. The majority of the upregulated genes were found to be downregulated upon long-term exposure to lower temperatures in chemostat experiments, where growth rate is constant. TORC1–Sch9, cAMP/PKA, and PKC1 pathways were found to be involved in the organization of the transcriptional response to heat shock and induction of the heat shock proteins was mediated by Hsf1p. High osmolarity glycerol (HOG) pathway was reported to be involved in the adaptation to cold stress that provokes changes in the membrane fluidity, which are sensed by Sln1p (Hayashi and Maeda, 2006; Panadero et al., 2006). However, the construction of quantitative models requires further studies as explained in the previous sections and remains as a challenging point.

**Table 3:** **Perturbation experiments to monitor the transcriptomic response of *S. cerevisiae* to the changing temperatures**.

Perturbation	Experiment type	Exposure (sampling) time	Measurement technique	Reference
**Heat shock or adaptation to higher temperatures**				
From 25 to 37°C	Shake flasks	5, 15, 30, and 60 min	cDNA microarrays	Gasch et al. (2000)
From 25 to 37°C	Shake flasks	0, 15, 30, 45, 60, and 120 min	Affymetrix YE6100 gene chips	Causton et al. (2001)
25 or 39°C	NA	20 min	Agilent Yeast Oligo Microarray	Ruiz-Roig et al. (2010)
From 28 to 41°C	Batch bioreactor	0, 10, 30, 60, 120, 240, and 360 min	Affymetrix Yeast Genome S98 Array	Mensonides et al. (2013)
From 30 to 42°C	Shake flasks	20 min	Agilent Yeast Oligo Microarray (V2)	Hughes Hallett et al. (2014)
**Cold shock or adaptation to cold**				
From 37 to 25°C	Shake flasks	5, 15, 30, 45, 60, and 90 min	cDNA microarrays	Gasch et al. (2000)
From 30 to 10°C	NA	15, 30, 120, 240, and 480 min	cDNA microarrays	Sahara et al. (2002)
From 30 to 10°C	Shake flasks	10, 30, and 120 min; some cases 60 h	DNA microarrays	Schade et al. (2004)
From 25 to 4°C	Batch/time series (up to 48 h)	6, 12, 24, and 48 h	cDNA microarrays	Murata et al. (2006)
30 and 12°C	Chemostat at 0.03 h^−1^	Sampling at steady state	Affymetrix Yeast Genome S98 Array	Tai et al. (2007)
30 and 15°C	Chemostat at 0.05 h^−1^	Sampling at steady state	Affymetrix Yeast Genome 2.0 Array	Pizarro et al. (2008)
28 and 15°C	Chemostat at 0.028 h^−1^	Sampling at steady state	Yeast Array	García-Ríos et al. (2014)

## Transcriptomic Response to Salt and Osmotic Shock

Cells exposed to increased osmolarity, leading to water loss and cell shrinking, need to maintain their shape and turgidity. For optimal functioning of biochemical reactions appropriate concentrations of ions are required in the cytosol and organelles. HOG pathway, which is a mitogen-activated protein kinase (MAPK) signal transduction system, was reported to be the major pathway in the adaptation of yeast cells to increased osmolarity by inducing glycerol formation (Hohmann, 2009). Osmostress induction was selected as a model system by several groups to understand the regulation of gene expression by stress-activated kinases and signal transduction. The high osmolarity signaling has been reviewed by Hohmann (2009) and de Nadal and Posas (2015). The detailed description of HOG pathway and its repressors were also extensively reviewed (Gehart et al., 2010; Saito and Posas, 2012; Engelberg et al., 2014). Several perturbation experiments were carried out to monitor the genome-wide transcriptomic response of yeast cells to the changing osmolarity in the environment.

The genome-wide analysis of the transcriptomic data of the wild-type and *HOG1* deleted yeast cells to saline stress created by the exposure to 0.4 or 0.8M NaCl for 10 or 20 min revealed that the genes involved in carbohydrate, glycerol, trehalose, and glycogen metabolism, the genes involved in the synthesis of ribosomal proteins, protein biosynthesis, and amino acid metabolism were induced after 10 min exposure to 0.4 or 0.8M NaCl. The genes associated with stress, signal transduction, and ion homeostasis were also found to be upregulated. The induction in the expression levels of the majority of genes was dependent on Hog1p. The examination of the trancriptomic response after 20 min indicated that this response was transient (Posas et al., 2000). Rep et al. (2000) analyzed the transcriptional response of the wild-type, *HOT1*, *MSN2 MSN4*, and *HOG1* deleted strains of *S. cerevisiae* to osmotic stress created by exposing cells to 0.5 or 0.7M NaCl or 0.95M sorbitol. This study has also revealed the upregulation of the similar set of genes that is reported by Posas et al. (2000), and the induction of the majority of them were found to be Hog1p dependent. Hot1p, which is now known as the transcription factor required for the genes involved in the synthesis of glycerol, was reported to be required for the normal expression of a set of genes of HOG pathway. The authors suggested that *MSN2/MSN4* might also be regulated by Hog1p.

Analysis of the dynamic transcriptomic response of *S. cerevisiae* cells grown to mid-log phase and treated with 1M NaCl for different periods including 0, 10, 30, and 90 min revealed that the number of salinity-induced genes increases over time. Early (10 and 30 min) transcriptional response genes were found to be involved in nucleotide and amino acid metabolism, intracellular transport, protein synthesis, and destination. A few components of signaling pathways were also found upregulated in this phase. Highly expressed transcripts identified after 90 min of the treatment included salinity stress-induced genes, transporters of the major facilitator superfamily, the genes involved in the metabolism of energy reserves, nitrogen and sulfur compounds biosynthesis, and lipid, fatty acid/isoprenoid biosynthesis. The genes involved in glycerol biosynthesis (*GPD1/2*, *GPP1/2*) were observed to be upregulated at all time points (Yale and Bohnert, 2001).

The analysis of the dynamic transcriptomic response of wild-type and strains that are blocked at various points in the HOG pathway, to various concentrations of KCl and 1M sorbitol revealed that Hog1p functions during gene induction and repression, cross talk inhibition, and in governing the regulatory period. Both branches of the HOG pathway were found to be active at high osmolarity and Ssk–Sln pathway has an important role in response to modest osmolarity (O’Rourke and Herskowitz, 2004).

Fine-tuning of the response to osmotic stress at translational level was also examined by several investigators. Melamed et al. (2008) investigated the translational response of yeast cells to high osmotic stress and its correlation with the transcriptomic response. This study revealed the accumulation of non-translated RNA corresponding to a set of genes. Most of the translationally regulated genes were found to be independent of the HOG pathway. The translational regulation of the HOG pathway-dependent genes was found to be mediated by Pub1p, and it has been suggested that the involvement of additional signaling pathways in the coordination of translational regulation. Analysis of the correlation with transcription and translatome, by monitoring the affinity tagged ribosomes, indicated that changes in the transcriptome are well correlated with translatome when the yeast cells were exposed to 1M sorbitol for 10 min to induce a severe osmotic stress and less correlated with the mild stress (Halbeisen and Gerber, 2009). Warringer et al. (2010) reported that the translationally regulated transcripts were dependent on Hog1p and Rck2p after hyperosmotic shock.

A yeast quantitative model of the Hog1 MAPK-dependent osmotic stress response was constructed by integrating immunoprecipitation data (ChIP-chip) with the transcriptome obtained from the analysis of single- and multiple-mutant strains (total of 31 different strains) exposed to 0.4M KCl for 20 min by Capaldi et al. (2008). This model revealed the interaction of Hog1 and Msn 2/4 pathways in information processing and regulation of gene expression in response to osmotic stress, which is context dependent. Chasman et al. (2014) have recently reported a very detailed integrative study on the pathway connectivity and the coordination of the signal in yeast cells in response to 0.7M NaCl. The transcriptomic data from 16 relevant mutants, which are carrier of the deletions in the genes known to be involved in the NaCl-induced acquired stress tolerance, was integrated with protein interaction data, and phospho-proteomic changes. This study shed light into the regulation and coordination of ESR genes and RNA Pol II was found to be key decision point in the coordination of balance between induced and repressed ESR. Cdc14p was found to be a critical integrator linking HOG and CK2 signaling, connecting to other pathways, including TORC1 and Ras/cAMP/PKA.

An integrative analysis carried out by Hughes Hallett et al. (2014), to investigate the effect of osmotic stress on the TORC1 pathway process information, revealed that the genes in the TORC1–Sch9 pathway were downregulated, and the expression levels of the genes in the TORC1–PP2A were not or little affected under osmotic conditions created with 0.4M KCl for a period of 20 min. TORC1–PP2A-branch signaling was weak, and Npr2p/Npr3p, Gtr1p/Gtr2p, and Rho1p and Snf1p were not involved in this signaling process. MAPK Hog1/p38 was found to be important in the inhibition of TORC1–Sch9 signaling, but not in other stress conditions caused by different perturbations of the system.

A comparative analysis of the transcriptomic response of two *S. cerevisiae* strains, a laboratory strain and a brewing strain, in response to high NaCl concentrations, revealed that the alterations in the expression levels of genes were larger in the laboratory strain. The response to the lower concentration of salt was rapid than that to the higher concentration in both strains. Under high NaCl concentration conditions, genes involved in carbohydrate metabolism and energy production were upregulated in both strains. Depending on the transcriptome profiles, target genes to construct a new strain with a better salt tolerance were identified, and the outcome of overexpression of those genes (*GPD1*, *ENA1*, and *CUP1*) was verified under high salinity stress (Hirasawa et al., 2006).

The details of the perturbation experiments in *S. cerevisiae* cells in response to salt and osmotic shock are summarized in Table 4. The cellular response of yeast cells to high osmotic perturbation was shown to be controlled by Hog1p, which also regulates the activities of Msn2p/Msn4p and TORC1 signaling pathway. Both branches of the HOG pathway were found to be active at high osmolarity, and Ssk–Sln pathway was shown to have an important role in response to modest osmolarity. A quantitative and explicit network model implicating pathway connectivity and coordination of signaling in response to osmotic stress was constructed using an integrative approach by Capaldi et al. (2008) and Chasman et al. (2014), respectively. However, the signaling dynamics and the identification of common and context-dependent features of oxidative response signaling remain to be elucidated.

**Table 4:** **Perturbation experiments to monitor the transcriptomic response of *S. cerevisiae* to salt and osmotic shock**.

Perturbation	Experiment type	Exposure (sampling) time	Measurement technique	Reference
0.4 or 0.8M NaCl	NA	10 and 20 min	DNA microarrays	Posas et al. (2000)
0.5 or 0.7M NaCl or 0.95M sorbitol	NA	45 min of 0.7M NaCl, 30 min of 0.5M NaCl, or 30 min of 0.95M sorbitol	Gene filters	Rep et al. (2000)
1M NaCl	NA	0, 10, 30, and 90 min	Gene filters	Yale and Bohnert (2001)
1M sorbitol or varying concentrations of KCl (0.0625, 0.125, and 0.5M)	Shake flasks	Sorbitol at 0, 5, 10, 20, 30, 40, 60, 90, 120, and 180 min	DNA microarrays (made in-house)	O’Rourke and Herskowitz (2004)
		KCl at 5, 10, 20, and 30 min		
1M NaCl	NA	0, 10, 30, 60, 180, and 300 min	DNA microarrays	Melamed et al. (2008)
1M sorbitol	NA	10 min	Oligonucleotide microarrays	Halbeisen and Gerber (2009)
0.4M NaCl	NA	0, 2, 6, and 30 min	Yeast 6.4K microarrays	Warringer et al. (2010)
0.4M NaCl	Shake flasks	20 min	Agilent G4140A microarrays	Capaldi et al. (2008)
0.7M NaCl	Shake flasks	30 min	DNA microarrays	Chasman et al. (2014)
from 30 to 42°C	Shake flasks	20 min	Agilent Yeast Oligo Microarray (V2)	Hughes Hallett et al. (2014)
0.25, 0.5, 0.75, and 1M NaCl	Shake flasks	0, 15, 30, 45, 60, and 120 min	Yeast Gene Chip ver. 2 (DNA Chip Research Inc., Japan)	Hirasawa et al. (2006)

## Transcriptomic Response to Perturbations in Metal Ion Homeostasis

Many essential cofactors in the cell are transient metal ions, and they are functional in a range of biological processes, such as cell energetics, gene regulation, and control of free radicals (Cohen et al., 2000). Although these cations are an essential part of nutrition, they are toxic at elevated levels, causing oxidative stress, or changes in enzyme and protein function, lipid peroxidation, and DNA damage. *S. cerevisiae*, one of the most intensively studied simple eukaryote, is a good model to study how the metabolism is affected by variations in response to the availability of metal ions due to the high degree of conservation among these mechanisms concerning yeast and other higher eukaryotes. There is a substantial amount of information regarding how *S. cerevisiae* deals with these metals and metalloids and copes with them at toxic levels (Wysocki and Tamás, 2010).

Among these metals, iron and copper are of particular interest due to their ability to donate and accept electrons in vital electron transfer reactions thus establishing themselves irreplaceable roles in many cellular processes (De Freitas et al., 2003). It also shed light on the pathophysiology of the related disorders of these processes in higher eukaryotes caused by the deficiency or the overload of these metal ions (Askwith and Kaplan, 1998).

### Response to Iron

Yeast cells respond to change in the iron availability by regulating the expression of iron transporters at transcriptional level. Aft1p was the first identified transcription factor that has a key role for the stimulation of the iron uptake systems in response to low-iron conditions (Yamaguchi-Iwai et al., 1995). Later, the genes regulated by *AFT2*, which is the paralog of *AFT1*, were identified using DNA microarrays. This study revealed that Aft2p encodes a transcription factor that has a role in the regulation of the expression in response to growth under low-iron conditions (Rutherford et al., 2001). Courel et al. (2005) designed the perturbation experiment to study the expression of genes involved in iron homeostasis by comparing the functions of Aft1p and Aft2p in regulation. Comparison of the transcriptome of the wild-type strain and corresponding *AFT1* and *AFT1 AFT2* deleted mutant cells, grown exponentially under iron-deficient conditions, revealed that Atf1p and its paralog Aft2p regulates the expression of genes related to iron-siderophore transport at the plasma membrane, vacuolar iron transport, and mitochondrial iron metabolism under iron-deficient conditions. Aft2p was identified to have a more specific role in the regulation of genes related to mitochondrial and vacuolar iron homeostasis, while Aft1p explicitly activates the expression of genes related to cell surface iron uptake systems.

Apart from the response observed in the iron ion transport to maintain the iron ion homeostasis, the genes involved in different metabolic pathways requiring iron-dependent enzymes were identified to be transcriptionally regulated in response to a change in the iron availability to remodel the metabolic activities for more efficient use of iron. Metabolic remodeling of yeast cells to iron deficiency was reviewed in detail by Philpott et al. (2012).

Shakoury-Elizeh et al. (2004) comparatively investigated the transcriptomic response of the wild-type and that of the mutant yeast cells (*AFT1* deleted or overexpressing Aft1p) grown in low (20 μM), iron sufficient (100 μM), and high (500 μM) iron conditions, until mid-exponential phase. In addition to the identification of novel target genes of Aft1p, biotin uptake was reported to be upregulated under iron deficiency, while cells preferred to synthesize it when iron is abundant. Similarly, many genes involved in the synthesis and uptake of amino acids that require Fe–S proteins were reported to be regulated by iron level. Regulation of glutamate synthesis was identified to be dependent on the iron availability. Its synthesis from ammonia and alpha-ketoglutarate was found to be catalyzed by the enzymes encoded by *GDH1* and its paralog *GDH3* under iron-deprived conditions. On the other hand, it was synthesized from glutamine and ketoglutarate under iron overloaded conditions through activity of *GLT1*, which was highly expressed. The integrative analysis of the transcriptome with metabolome data of the yeast cells grown in the presence of low (10 μM), optimal (100 μM), and high (330 μM) concentration of iron for 4 h revealed that the glucose metabolism, amino acid synthesis, ergosterol, and lipid biosynthesis biological processes were all affected due to the loss in the activities of specific iron-dependent enzymes under iron deprivation. However, the amino acid homeostasis was not found to be very much affected from iron deficiency. Iron uptake systems were upregulated to preserve the activity of the iron-containing enzymes. It has been suggested that yeast cells do not have a specific machinery to forward iron ions to be used in a specific process under iron deficiency (Shakoury-Elizeh et al., 2010).

Puig et al. (2005) studied and compared the transcriptional response of yeast cells grown to the exponential phase under iron deprivation condition achieved by the addition of iron chelator or in 300 μM Fe^1+^-containing medium. The gene encoding fatty acid desaturase and genes related to sterol biosynthesis were upregulated, and the genes associated with TCA cycle, mitochondrial electron transport chain, heme biosynthesis, and biotin synthesis were downregulated under iron-deprived conditions. The same group further studied the role of *CTH2*, which was identified to be specifically induced under iron-deprived conditions, in iron regulon. The transcriptional response of the *CTH2* and *CTH1 CTH2* deleted mutants to iron deprivation indicated that Cth2p is involved in the targeted degradation of the transcripts coding for proteins involved in multiple Fe-dependent metabolic pathways, including the TCA cycle, respiration, lipid metabolism, heme biosynthesis, and multiple Fe–S proteins. It has been suggested that metabolic remodeling in response to iron deprivation is coordinated through targeted degradation of mRNAs encoding proteins, which are involved in Fe-dependent processes and mediated by Cth2p. Further studies conducted by the same group (Puig et al., 2008) identified Cth1p having also an important role in the targeted degradation, specifically mRNAs encoding proteins involved in mitochondrial oxidative phosphorylation, while Cth2p preferentially involved in the targeted degradation of mRNAs of iron-containing enzymes and mRNAs associated with iron homeostasis. These two proteins were also reported to have important roles in the degradation of mRNAs involved in the transport and metabolism. An increase in the glycogen level and activation of Snf1p was also observed in response to iron deficiency.

The analysis of the transcriptional response of the wild-type and iron sensitive *CCC1* deleted cells to high levels of iron under aerobic and anaerobic conditions, grown in the presence of 3 mM iron for 3 h, revealed that high iron alters the expression of the genes involved in cell-cycle progression, DNA repair, and oxidative response and iron toxicity in the *CCC1* deleted cells. The same study also revealed that iron toxicity may not be only due to oxidative damage since the increase in the transcripts indicative of oxidative damage or DNA repair in response to high iron levels was not observed in the cells under anaerobiosis. Iron sensitivity caused by the deletion of *CCC1* was reported to be suppressed by the upregulation of the genes encoding mitochondrial iron transporters Mrs3p or Mrs4p or mitochondrial pyrimidine phosphate transporter Rim2p. In this study, it was suggested that cells may decrease cytosolic iron levels by a mechanism that sequester iron ions into mitochondria under iron toxicity (Lin et al., 2011).

In order to investigate the role of the Yap5p transcription factor in the transcriptional reorganization of yeast cells, the transcriptomic response of the *YAP5* deleted cells to iron overload was examined after 20 and 60 min exposure to 2 mM FeSO_4_ and compared with that of the wild-type yeast cells (Pimentel et al., 2012). In addition to the alterations in the expression levels of the genes involved in iron homeostasis, this study revealed that the genes involved in ribosome biogenesis were downregulated and those of involved in stress response, protein degradation, respiration, lipid, fatty acids, and carbohydrate metabolism were upregulated under high iron-containing conditions, indicating that iron overload causes a GSR. Yap5p was found to be involved in the regulation *GRX4*, which regulates the nuclear localization of Aft1p, and the expression of *CCC1*, which is involved in iron storage, was found to be partially regulated by Yap5p. A schematic model to illustrate the hypothetical role of Yap5p under iron overload was also proposed by the authors. The regulation of iron metabolism by low and high iron sensing transcription factors (Aft1p/Aft2p and Yap5p) and post-transcriptional regulation of iron metabolism was reviewed by Outten and Albetel (2013).

### Response to Copper

The transcriptional response to low and high copper levels was reported to be regulated by transcription factors Mac1p and Ace1p, respectively (Jungmann et al., 1993). Gross et al. (2000) investigated the genome-wide transcript profiles under copper deprived and excess conditions by exposing the cells to 100 μM CuSO_4_ for 30 min, to identify the targets of Mac1p and Ace1p. These experiments revealed that Mac1p activates the expression of *CTR1*, *CTR3*, *FRE1*, *FRE7*, YFR055w, and YJL217w under copper deficient conditions. Under copper overloaded conditions Ace1p induces the expression of the genes encoding metallothionein that chelates excess copper, Cup1p and Crs5p, and cytosolic copper–zinc superoxide dismutase, Sod1p, which detoxifies superoxide. The expression of the two genes that have roles in iron uptake system, namely *FET3*, which is required for high-affinity iron uptake, *FTR1* which forms complex with Fet3p, were also found to be downregulated under high copper conditions (Gross et al., 2000).

The genome-wide time-course gene expression analysis during copper starvation and copper overload in the presence of 8 μM CuSO_4_ for a period up to 48 h highlighted the connection between copper and iron metabolism. In response to copper deprivation, cells induce the expression of copper-independent, non-reductive iron transport genes although the global cellular iron levels did not decrease. Similar to the response observed under iron-deprived conditions, copper deficiency also lead to downregulation of the genes involved in respiration (van Bakel et al., 2005).

Cankorur-Cetinkaya et al. (2013) compared the transcriptional profiles of the wild-type and *CCC2* deleted cells under copper deficient and high or low levels of copper-containing conditions by growing yeast cells without copper or 0.04 μM or 0.5 mM CuSO_4_. *CCC2* is the human ortholog of human *ATP7A* and *ATP7B*, in which mutations are the cause of Menkes and Wilson diseases, respectively. Experimental design used in this study enabled the identification of the genes and biological processes affected from the deletion of the *CCC2* gene or the changing extracellular copper levels or the interactive effect of both factors. This study also showed the relation between copper and iron metabolisms highlighting the alteration in the transcriptional response to different level of copper availability in the absence of *CCC2*, which is involved in the copper export from the cytosol. Ribosome biogenesis and copper import were found to be downregulated in reference yeast cells in response to changes from low/deficient copper condition to high copper condition. This study revealed the processes, regulation of which under different copper levels changes depending on the presence or absence of *CCC2*. The genes involved in iron ion homeostasis, siderophore transport were identified to be upregulated in the reference strain, whereas downregulated in the absence of *CCC2* deleted cells in response to high copper levels. The iron homeostasis, siderophore transport, and NAD^+^ metabolism were identified to be downregulated in the deletion mutant under high copper-containing conditions and upregulated under copper-deficient conditions when compared to the reference strain under same conditions. The amino acid metabolism, specifically arginine metabolic process, was also identified to be altered by the interactive effect of both perturbations, and these findings were also supported by the metabolomic analysis. The transcription factors, around which most transcriptomic changes occur, were also identified in this study through integrative analysis of transcriptome and regulome. This integrative analysis indicated that the genes targeted by general oxidative stress inducers, namely Sko1p, Skn7p, Cin5p, Yap1p, and Yap6p, were mostly affected from both perturbations and their interactions.

### Response to Other Metal Ions

The transcriptional response of yeast to zinc deficiency was studied in glucose- and ammonium-limited chemostat cultures aerobically and anaerobically (De Nicola et al., 2007). Zinc-specific Zap1p regulon, a central transcription factor that is active in response to zinc alterations, was identified to be the regulator of genes involved in carbohydrate storage metabolism. It was found that oxygen and Zn availability affected a large number of genes, implying a more significant role of Zn in mitochondrial processes. Wu et al. (2008) used transcriptome profiling of the wild-type and mutant cells, *ZAP1* deleted and cells containing plasmids encoding constitutive allele of Zap1, to detect the target genes of Zap1p. Yeast cells were exposed to various concentrations of zinc changing from 3 to 300 μM for various periods of time changing from 0.5 to 8 h. In addition to their previous study (Lyons et al., 2000), in which 46 genes were identified to be potential target genes; in this study, they further investigated the role of Zap1p and identified numerous new targets of Zap1-mediated regulation. The transcriptomic response of the Zap1 target genes was shown to have dependency to the level of zinc in the medium. This study revealed that cells induce the genes involved in zinc uptake to maintain the zinc homeostasis in response to a mild zinc deficiency. On the other hand, the genes involved in maintaining secretory pathway and cell wall function, and stress responses were regulated at transcriptional level in response to a severe zinc deficiency. It has been suggested that these group of genes are mainly involved in the adaptation to zinc deficiency.

Jin et al. (2008) investigated the genome-wide response of yeast cells to a 2 h of exposure to equitoxic concentrations of Zn^2+^, Cd^2+^, Hg^2+^, Cu^2+^, Ag^+^, Cr^6+^, and As^3+^ and complemented this study with deletome. Principal component analysis (PCA) and hierarchical clustering of global transcriptomic response indicated that the response was specific for each metal and the response to different concentrations of the same metal were closely related but different. A group of genes, called common metal responsive (CMR) genes, were found to be commonly affected by all metals. The genes involved in metal transport and homeostasis, detoxification of ROS, carbohydrate metabolism, including glycolysis, oxidative phosphorylation, and alcohol metabolism, polyamine transport, and transcription were upregulated. The genes involved in polysaccharide metabolism, G-protein signaling, protein targeting, and transport were downregulated. Some evolutionarily conserved, signal transduction pathways, including cAMP-dependent PKA, protein kinase CK2, and MAPK, were found to be involved in the regulation of responses to the exposure to metals. Msn2p/Msn4p was observed to regulate 10% of the differentially expressed genes, among the 14 perturbation conditions.

A short-term effect of the moderate amounts of metals on *S. cerevisiae* cells was investigated (Hosiner et al., 2014). Analysis of the transcriptomic data after 30 min acquaintance to Ag^+^, Al^3+^, As^3+^, Cd^2+^, Co^2+^, Hg^2+^, Mn^2+^, Ni^2+^, V^+^, and Zn^2+^, metals relevant to human health indicated that the metal-specific oxidative defense, including gluthation/thioredoxin and metallothionein systems, and protein degradation processes, including vacuolar protein degradation, proteosomal proteolysis, chaperone complex activities, and Sec19, which regulates vesicle traffic in secretory pathways, were activated in response to all these metal ions. The genes involved in ribosome biogenesis were observed to be downregulated after a short-term exposure to metals. The potential regulators effective under these conditions were predicted *via* a statistical tool, and the largest group covered the transcription factors Yap1p, Msn2p, Msn4p, Yap7p, and Cad1p, the latter two being the homologs of Yap1p. A comparative analysis with the results obtained after 2 h exposure to metals (Jin et al., 2008) showed the induction of the genes involved in protein synthesis and a repression of the genes associated with the metal detoxification process.

The details of all these perturbation experiments in *S. cerevisiae* cells are summarized in Table 5. All these studies indicated that yeast cells reorganize their transcriptional response and their metabolism upon exposure or deficiency of transition metals.

**Table 5:** **Perturbation experiments to monitor the transcriptomic response of *S. cerevisiae* to changes in metal ion homeostasis**.

Perturbation	Experiment type	Exposure (sampling) time	Measurement technique	Reference
**Response to iron**				
Deletion or overexpression of AFT2	Shake flasks	Sampled at OD = 0.4	DNA microarrays	Rutherford et al. (2001)
*aft1*Δ and *aft1*Δ*/aft2*Δ cells grown in iron depletion	NA	Sampled at log phase	DNA microarrays	Courel et al. (2005)
Deletion or overexpression of AFT1 under 20, 100, or 500 μM iron-containing conditions	NA	Sampled at mid-log phase	cDNA microarrays	Shakoury-Elizeh et al. (2004)
at mid-exp. phase switch from 100 to 10, or 300 μM iron-containing conditions	NA	4 h	Affymetrix Yeast Genome 2.0 Array	Shakoury-Elizeh et al. (2010)
No iron or 300 μM iron-containing conditions	NA	Sampled at log phase	DNA microarrays	Puig et al. (2005)
*cth1*Δ*/cth2*Δ cells expressing physiological levels of *CTH1*, *CTH2*, or vector alone under iron-deficient conditions	NA	Sampled at log phase	Affymetrix Yeast Genome S98 Array	Puig et al. (2008)
*ccc1*Δ exposed to 3 mM of iron under aerobic and anaerobic conditions	NA	3 h	Affymetrix microarray	Lin et al. (2011)
2 mM iron	Shake flasks	20 and 60 min	UAVR Yeast 19.2K v1	Pimentel et al. (2012)
**Response to copper**				
Wild-type versus constitutively active Mac1 at mid-exp. phase switch to no copper, or 100 μM copper containing conditions	NA	60 min of no copper	DNA microarrays	Gross et al. (2000)
		30 min of 100 μM CuSO_4_		
At mid-exp. phase switch to no copper, or 8 μM copper containing conditions	NA	0.5, 1, 2, 3, 4, 24, and 48 h	A-UMCU-4 – UMC Utrecht *S. cerevisiae* 16K array, version 1.2	van Bakel et al. (2005)
Wild-type versus *ccc2*Δ*/ccc2*Δ	Batch bioreactor	Sampled at mid-log phase	Affymetrix Yeast Genome 2.0 Array	Cankorur-Cetinkaya et al. (2013)
No copper, 0.04 μM or 0.5 mM copper containing conditions				
**Other metal ions**				
Zinc limitation at aerobic and anaerobic conditions	Chemostat at 0.1 h^−1^	Sampled at steady state	Affymetrix Yeast Genome S98 Array	De Nicola et al. (2007)
Zinc replete (various Zn concentrations) and zinc limiting	NA	At log phase or transient: 0.5, 1, 2, 4, and 8 h	DNA microarray	Wu et al. (2008)
Equitoxic concentrations of Zn^2+^, Cd^2+^, Hg^2+^, Cu^2+^, Ag^+^, Cr^6+^, and As^3+^	NA	2 h	DNA microarray	Jin et al. (2008)
Ag^+^, Al^3+^, As^3+^, Cd^2+^, Co^2+^, Hg^2+^, Mn^2+^, Ni^2+^, V^+^, or Zn^2+^	Shake flasks	30 min	Microarrays obtained from Microarray Centre Toronto	Hosiner et al. (2014)

Since the iron deficiency is the most common, worldwide nutritional disorder, first perturbation experiments related to iron were designed to understand the effect of the iron deficiency on the reorganization and the regulation of transcriptomic response. These studies resulted in the identification of the target genes controlling iron transport and homeostasis as well as the post-transcriptional regulation of the iron-dependent metabolic pathways under iron deficiency. Aft1p, Aft2p, Yap5p, and Snf1p were found to be the major transcription factors in regulation of the cellular reorganization under iron deprivation.

Although a number of experiments were also carried out to understand the effect of the iron overload, the results of these experiments are not comparable due to the use of different iron concentrations and exposure times. A set of carefully designed perturbation experiments should be planned to understand the effect of the iron overload and to reveal the signaling pathway(s) underlying the response to iron overload and deficiency.

Early copper-related genome-wide studies are concentrated on the identification of target genes of two transcriptional factors in response to copper deficiency and copper overload. However, these pioneering studies are not easily comparable due to the differences in experimental design as in the case of iron.

The perturbation experiments carried out by exposing yeast cells to low or high levels of different metal ions for a defined period of time revealed a detailed picture for the organization of the transcriptional response to metals, including copper. Evolutionarily conserved signaling pathways, including cAMP-dependent PKA, protein kinase that coordinate the post-translational regulation of the proteins involved in the glycolysis and gluconeogenesis and the transcriptional regulation of the synthesis of ribosomal protein, CK2 that is involved in ribosome biosynthesis, and MAPK that is involved in apoptosis, differentiation, and stress response were identified to participate in the organization of the response to metals. Msn2p/Msn4p, Yap1p, and its homologs Yap7p and Cad1p were found to be involved in the regulation of a large number of metal responsive genes. However, a quantitative model implicating these results is still missing.

## Conclusion and Perspective

Yeast cells may encounter many kinds of environmental perturbations during growth and fermentation, including accumulation of ethanol, weak acids, heat, low pH, ROS, nutrient limitation, and osmotic changes imposed by high concentrations of sugars (Teixeira et al., 2011; Zhao et al., 2015). In most cases, cells have to cope simultaneously or successively with numerous aspects. Studying these responses is important for optimizing industrial process conditions and designing more robust overproduction strains using a rational design strategy and genetic engineering techniques. In addition to that, such perturbation/response studies are important due to their contribution to our fundamental understanding of microbial metabolism and to unravel drug-disease relationships. Here, we summarized findings on the yeast transcriptome in response to nutritional, osmotic, oxidative, temperature, and transient metal ion perturbations, as such not aimed for an exhaustive literature survey.

Transcriptomic analysis of *S. cerevisiae* subjected to different types of stress-causing conditions helped us to identify the genes with altered expression as a result of a perturbation and the use of deleted strains for the known transcription factors led to the determination of the affected genes and the up- or downregulated biological processes. However, studies carried out in the first decade since the application of the high-throughput techniques have discrepancies due to the differences in experimental design including the type of the equipment or microarrays, fermentation conditions and mode, selection of control, duration of the exposure to stress-causing agents, and the amount of stress-causing agent. The first perturbation experiments were carried out in batch. Chemostat fermentations were preferred after the elucidation of the relationship between the response and the growth rate. The statistical and bioinformatics tools used to analyze the results are also important features that make the comparative analysis very difficult.

In the last decade, carefully designed perturbation experiments considering prior genetic data and the integration of transcriptome with metabolome, proteome, phospho-proteome, and interactome revealed the presence of some shared signaling pathways, which are activated in response to several environmental perturbations and also the condition dependent response of *S. cerevisiae* to specific perturbations. Ras/cAMP/PKA signaling pathway, which is involved in cell growth and response to nutrients and stress, and TORC1, which is the main regulator of growth and metabolism in all eukaryotic cells, were found to be common in response to all perturbations considered in this review. Sch9-branch signaling and PP2A-branch signaling of TORC1 were reported to be inhibited under glucose starvation, osmotic stress, oxidative stress, and heat stress in yeast cells. Different types of nitrogen starvation and rapamycin were observed to lead to the activation of PP2A-branch signaling of TOR kinase (Hughes Hallett et al., 2014). Snf1p, AMP-activated serine/threonine protein kinase, which is involved in the transcription of glucose repressed genes in yeast, is also a common regulator in organizing the response of yeast cells to several perturbations. In addition to the common signaling pathways, ESR genes, primarily controlled by the transcription factors Msn2p and Msn4p, which are known to be regulated by Ras/cAMP/PKA pathway, and/or by TORC1, are also induced in response to various environmental perturbations. However, the role of the Msn2p and Msn4p for the acquisition of stress tolerance has remained rather obscure. While Berry and Gasch (2008) suggested a significant role of Msn2p and Msn4p in the gain of stress tolerance, Zakrzewska et al. (2011) claimed that these two transcription factors are not functional for the acquisition of severe stress tolerance, but decline in the growth rate is critical for the gain of tolerance. However, the molecular mechanism leading to the development of tolerance, which is of extreme importance for biotechnological applications, requires further examination.

The major goal of system biology is to construct quantitative whole life models for the prediction of cellular response to the changing environment. When we overview the results of a number of studies carried out in the last 15 years, which are focused to elucidate the response to an induced stress using one or several omics technologies, it seems that we are far away to reach this aim at present. However, it should be noted that the efforts, especially on the development of integrative systems biology approaches complemented with accumulated and shared information, continue and guarantee the success in the near future.

### Future Prospects

**Figure 1:**
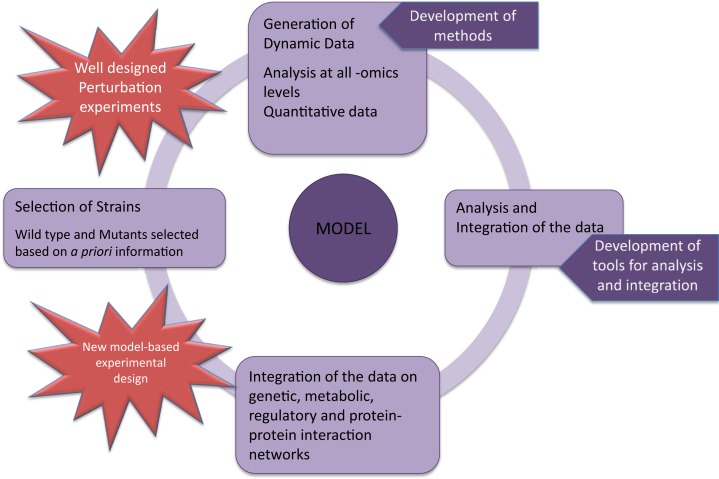
**Systems biology-based strategy for the construction of whole-genome models in *S. cerevisiae***.

Yeast cells have established a range of mechanisms in response to environmental and genetic perturbations, in order to adopt themselves to the new condition. This is attained by not only changes in the gene expression levels and protein regulation but also mRNA stability, non-covalent binding of allosteric effectors and post-translational modifications of enzymes are involved (Tripodi et al., 2015). Therefore, despite the large number of perturbation experiments carried out to date, understanding the complete regulatory and signaling networks in response to perturbations requires further carefully designed perturbation experiments complemented with integrative analysis of -omics and new computational approaches (see Figure 1). It should be noted that strain selection and selection of the mutants, which will be incorporated into the study should be carefully planned by incorporating prior genetic knowledge. The complementation of environmental perturbations with genetic perturbations using appropriate mutants will help to characterize the details and coordination of the underlying signaling and regulatory events.

Most of the reviewed articles here used microarray technology for studying transcriptome. Since the RNA-seq technology has been available for several years now, the number of studies employing this technology is expected to accumulate and play a major role in future research. The properties of RNA-seq such as providing more precise measurement of transcript levels, and excluding limitations such as non-specific hybridization and possible signal saturation due to high abundance of transcripts makes it a valuable tool (Wang et al., 2009; Nookaew et al., 2012).

Most of the studies carried out in the past on the yeast transcriptome comprise measurements at single-type point (static). Time-series transcriptome data complemented with quantitative proteome and phospho-proteome reflect the dynamic regulation of gene expression, thus incorporation of such data improves the validity of a quantitative model, derived to predict the activities of genes under a particular condition. The design of new perturbation experiments to monitor dynamic changes at all -omics levels including interactome and metabolome and the development of new computational tools to analyze and integrate this data will possibly facilitate to reveal the underlying regulatory and signaling events and will immensely contribute to improve the predictive capability of models in the near future.

It was not within the scope of this review, but it is noteworthy that the yeast response mechanisms to cope with the presence of a foreign compound, i.e., drug, is an attractive research field to discover drug targets or understand the mechanism of action. Studies on the deletion or overexpressing mutants are considered very important in the understanding of molecular basis of diseases and in finding novel drug targets. For example, the MAP kinase Hog1 is the yeast ortholog of mammalian p38, important for embryonic development and cancer progression (Bradham and McClay, 2006). Consequently, yeast will continue to be one of the major model organisms for systems biology approaches and specifically designed perturbation experiments will help to develop whole life models.

## Author Contributions

All authors participated equally in the preparation of this contribution, have read, and approved the final manuscript.

## Conflict of Interest Statement

The authors declare that the research was conducted in the absence of any commercial or financial relationships that could be construed as a potential conflict of interest.
